# Lumbar muscles biomechanical characteristics in young people with chronic spinal pain

**DOI:** 10.1186/s12891-019-2935-z

**Published:** 2019-11-23

**Authors:** Wai Leung Ambrose Lo, Qiuhua Yu, Yurong Mao, Wenfeng Li, Chengpeng Hu, Le Li

**Affiliations:** 1 Department of Rehabilitation Medicine, The First Affiliated Hospital, Sun Yat-sen University, Guangzhou, 510080 China; 2 Guangdong Engineering and Technology Research Center for Rehabilitation Medicine and Translation, The First Affiliated Hospital, Sun Yat-sen University, Guangzhou, 510080 China

**Keywords:** Muscle tone, Muscle stiffness, Paraspinal muscle, Low back pain, Myotonometer

## Abstract

**Background:**

The prevalence of low back pain is rising among the young adult population. Altered lumbar muscle tone was suggested to be associated with underlying pathologies and symptoms. To date, there is minimum information available on the repeatability of lumbar spine muscle mechanical properties in the young adults who experienced low back pain. This study aimed to assess the reproducibility of mechanical properties of lumbar spinal muscle in young adults with spinal pain by myotonometer and explored the difference in reproducibility when different number of indentations was used.

**Methods:**

Participants who aged between 18 to 25 and reported chronic LBP were recruited. Lumbar muscle tone (Hz) and stiffness (N/m) were assessed by myotonometer on one occasion by two assessors. Parameters were recorded by triple scans and 5-scans mode. Intraclass correlation coefficient (ICC), standard error of measurement (SEM), smallest real difference (SRD), Bland and Altman analysis were used to assess agreement between two measurements. The relationship between muscle mechanical properties and pain score and disability level were assessed by Spearman’s rank correlation coefficient.

**Results:**

The results of ICCs indicated excellent repeatability in triple scans and 5-scans mode for each lumbar level bilaterally (ICC > 0.75). SEM and SRD were smaller in triple scans than 5-scans mode for most levels. Bland and Altman analysis revealed no systematic bias. Spearman’s rank correlation analysis indicated significant high correlations between muscle tone and disability level (*r* = 0.80, *p* < 0.05), and between muscle stiffness and disability level (*r* = 0.81, *p* < 0.05).

**Conclusions:**

This study found that lumbar spinal muscle tone and stiffness were repeatable parameters when measured by myotonometer. The reproducibility of muscle mechanical parameters did not appear to differ between the two scanning modes with different number of indentations. Muscle tone and stiffness measured by myotonometer may therefore be reliable as outcome measures to assess intervention induced changes. The lack of significant association between intensity of pain and mechanical properties of paraspinal muscles may suggest that muscle properties measured at rest might not be related to pain level at rest but more related to pain elicited during movement.

## Introduction

The Global Burden of Disease Study indicated that low back and neck pain were among the top four conditions that led to disability [1]. Prevalence of low back pain (LBP) among young adults is on the rise with a prevalence rate reported to be 42.4% [2]. Altered lumbar muscle tone was found in adults with chronic LBP [3, 4] and was suggested to be associated with underlying pathologies and symptoms [5]. Muscle tone can be defined as the resting tension or the resistance in response to stretch [6]. Early literature indicated that muscle tone is a contributing factor to the pain-spasm pain model [7] that suggest that pain results in increased muscle tone which in turn causes pain [8]. The pain adaptation models postulates that pain itself reduces activation of muscles which reduce range of motion and movement velocity This in turn leads to appropriate body positioning at rest and during motion. Muscle tone comprises of both neural and non-neural components [3]. The neural component include the tonic stretch reflex that is facilitated by the neural commands originated from cortical and subcortical centres, spinal circuitry and peripheral input. The non-neural component is related to the viscoelastic properties of muscle tissues that is related to multiple elements within the musculotendinous unit such as protein and collagen [9].

Alteration of muscle tone may induce pain, inadequate movement control and posture. Existing literature suggests that high muscle tone may be involved in the pain-spasm-pain vicious circle that contributes to chronic pain [10]. Intervention such as mechanical indentations therapy [3] spinal manipulation and mobilization are often used as means to reduce pain by altering muscle tone. Clinical decision of intervention type and treatment effectiveness are guided by the results of clinical assessments such as feeling the soft tissue resistance during passive elongation via passive joint motions [11], or by palpatory technique such as manual spinal stiffness assessment [12]. These techniques mostly concern facet joint motion stiffness and have less focus on the mechanical muscle properties. Other authors suggested the use of subjective palpation to identify altered muscle firmness and rigidity to elicit pain [13]. Published studies have criticized the poor reliability of manual technique to assess mechanical muscle properties of tone and stiffness [14, 15]. Electromyography (EMG) is a common mean to assess muscle tone in laboratory setting [16]. One of the limitation of using electrical activity level as a surrogate for muscle tone is that it mostly concerns the muscle contraction elicited by neural electrical drive of motor nerves and muscle cells but not the endogenous contractile structures of skeletal muscles. The non-neural component of muscle tone that is related to the mechanical muscle properties occurs without the electrical activity in the muscle cell [9]. The development of shear wave elastography technique offers a quantifiable spatial representation of the viscoelastic characteristics of skeletal muscle [17]. Shear wave elastography assesses muscle stiffness by the analysing the propagation velocity of the induced shear waves recorded from a defined region of interest in the targeted muscle and tendon [18]. The interpretation of shear wave elastography data relies on radiologist with specialised knowledge with muscular biomechanical concepts and technical operation of the assessor [17]. In addition, access to shear wave elastography equipment is often limited to large hospital or research institute due to its acquiring and maintenance cost [18].

Early literature suggested that indentation device such as myotonometer that applied a known amount of pressure to the muscle and measured the resonance of muscle fiber oscillation was a potential way to quantify mechanical muscle properties of tone and stiffness [19]. The validity of the myotonometer was investigated in several studies [20–22]. The majority of the validation studies were conducted with previous models of handheld myotnomoter. Findings of the validation studies suggested that myotonometer may identify substantial changes in spastic elbow muscle [22] and differentiate abnormal muscle tone between patients with ankolyinsing spondylitis and healthy individuals [23]. Muscle tone and stiffness were also found to be positively correlated with lower limb muscle strength and thickness [18] and upper limb hand motor function [21, 24]. A validation study indicated that the stiffness of the erector spinae at rest measured by elastography was moderately correlated with muscle stiffness measured by myotonometer. Changes in erector spinae stiffness as measured by myotonometer at different contraction intensities were also comparable with stiffness measured by elastography [25]. Another study concluded that the surface EMG activity is concurrent with the extensor myofascial tone [4]. Recently published studies with the latest model of handheld myotonometer suggested stiffness measured by handheld myotonometer was significantly correlated with the stiffness measured by shearwave elastography [18]. Another study also reported less tissue resistance at the convex side than the concave side in people with idiopathic scoliosis [26]. To date, there is minimal information regarding the mechanical muscle properties of tone and stiffness in young adults with chronic LBP. It is also unclear if the mechanical muscle properties of lumbar spinal muscles are repeatable parameters that could potentially be used as an outcome measure when recorded in a clinical setting since mechanical muscle properties may be affected by the background noise that is present within the clinical environment [6]. There is also uncertainty on the optimum number of indentation to be used without compromising time effectiveness and reliability. A study that was conducted in the healthy population suggested that a single indentation was sufficient to provide valid and reliable measurement [4]. Whereas, a study conducted on elderly with paratonia indicated a higher number of indentation would yield more reproducible average measurement when compared with lower number of indentation [6]. To date, it is unknown if the number of indentation may have any influence on muscle properties reproducibility in young people with chronic LBP since the indentation may stimulate the stretch reflex response.

This study aimed to assess the reproducibility of the measures of paraspinal muscle tone and stiffness in young adults with chronic LBP when recorded in a clinical environment on one occasion. The study also explored the difference in the reproducibility of parameters when different number of indentations were used to record muscle tone and stiffness.

## Methods

### Study setting

The study took place in the rehabilitation department of The First Affiliated Hospital, Sun Yat-sen University. Biomechanical properties of paraspinal muscles were recorded while the participants lied in prone on a height-adjustable couch within the treatment area.

### Recruitment

The student and staff population of the local institute were recruited as participants via displayed poster, social media and word of mouth. Interested participants were provided with participant information sheet. All participants were encouraged to ask questions regarding the study. Eligibility screening was conducted by clinical staff or a member of the research team.

### Sample population

The study had the following inclusion criteria: 1) age between 18 to 25; 2) reported to have consistent local pain between L3 and the gluteal fold for a minimum of 6 weeks prior to study enrollment [27]; 3) did not receive treatment for more than 2 weeks prior to study enrollment; 4) have at least “minimum impact on activity” as assessed by the Osewstry Disability Index. Diagnosis of low back pain was conducted by a physical therapist following the clinical assessment protocol published by the American College of Physicians and the American Pain Society [28]. During the literature search, it was found that a wash out period that ranged between 1 day [29] and 4 weeks [30] were adopted in cross over trial that involved participants with low back pain. Thus this study chose the average period of 2 weeks as a wash out period.

This study had the following exclusion criteria: 1) body mass index (BMI) classified as “overweight” [31]; 2) history of spinal pathology, neurological disorders and presence of co-morbid conditions; 3) presence of open wound around the recording site.

### Ethics

The study protocol was reviewed and approved by ethical committee of the First Affiliated Hospital, Sun Yat-sen University, China [approval no.: 2016(85)]. The declaration of Helsinki was strictly adhered to throughout the study. Written informed consent was obtained from all participants. Participants were informed that they were free to leave the study at any point without giving a reason.

### Instrument

MyotonPRO® (Estonia) was used to record lumbar spinal muscle tone and stiffness. A pre-compression force of 0.18 N was first applied to the skin by pressing the test probe vertically against the tested muscle. Once the pressure was reached, the device then applied consecutive indentations with a force of 0.4 N (15 ms apart) to generate damped oscillation within the muscle fiber. Muscle tone and stiffness were calculated from the oscillation pattern induced by the indentations. Muscle tone was calculated as the natural oscillation frequency (Hz) as 1/T, where T is the duration of oscillations measured in second. The transducer records the maximal acceleration of oscillation and the deformation of the tissue to derive muscle stiffness (N/m) [32]. This study employed triple scans and 5-scans mode. In tripe scans mode the, the device applied 3 consecutive indentations, 0.8 s apart. The device applied 5 consecutive indentions in 5-scans mode. The calculation of tone and stiffness by the device is documented in several publications [32–34].

### Procedure

Demographics data and clinical information were recorded at the beginning. Participants were asked to score their average level of pain over the past 6 weeks and rate on a 0–10 Numerical Pain Rating Scale (NPRS). Participants lied in prone on an examination couch, exposing the lumbar spine. The test sites were identified in accordance to a method previously proposed (Nair et al., 2016). The superior border of the iliac crests was first palpated to estimate the intervertebral space between of L_3_ and L_4_. The spinous processes of L_3_ to L_5_ were subsequently located and marked. Participants were then asked to perform a low level of isometric extension sufficient enough to reveal the extensor muscle bulk prominences. The test sites were marked as the same level as the spinous process of L_3_ to L_5_. Participants placed their hands beside head and lied in prone. Measurements were recorded first on the left, then progressed to the right side in the order of L_3_ to L_5_. Participants performed a 5-s breath hold at the end of the inspiration phase. Measurements were taken during the breath hold period to minimize confounding factor on muscle properties that was related to intra-abdomen pressure change. Measurements were recorded by two assessors in the same order. A 15 min break was provided after each complete series was recorded.

### Data analysis

The software SPSS 20 (IBM, Armonk, New York, US) was employed to conduct statistical analyses. Kolmogorov-Smirnov test and frequency histograms were first conducted to confirm data normality. The sample population characteristics were assessed by descriptive statistics. The intraclass correlation coefficient (ICC) model 3, k was adopted to assess the repeatability of muscle tone and stiffness. The interpretation of ICC levels was as follow: Excellent> 0.75; Good to Fair = 0.74–0.40; Poor < 0.40 [35]. Standard error of measurement (SEM) [36], smallest real difference (SRD) [37], Bland and Altman analysis and 95% limits of agreement (LOA) [38] were used to assess the agreement between two measurements.

The relationship between muscle mechanical properties and pain score and disability level were assessed by Spearman’s rank correlation coefficient.

## Results

### Sample population

This study enrolled fifteen participants with chronic LBP. The mean age of the cohort was 21.8 years old (7 males, 8 females). All participants were able to complete the experiment protocol. Numerical Pain Rating Scale range between 3 to 6 (mean 4 ± 1). Table 1 presents the characteristics of the cohort. The pain location, numerical pain rating scale and mean muscle tone and stiffness of L_3_ to L_5_ stiffness are presented in Table 2.

**Table 1:** Characteristics of the sample population

Basic information	
Age	21.80 (0.9)
Body Mass Index	21.0 (3)
Dominant side	All right
Male/Female	7/8
Clinical information	
Number Pain Rating Scale	4 (1)
Pain location (left/right/bilateral)	5/3/7
Owstery Disability Index	5.3 (2.4)

**Table 2:** Mean muscle tone and stiffness according to pain location of each participant. Key: NPRS-numerical pain rating scale

Pain location	NPRS	Left		Right	
		Muscle tone (Hz)	Stiffness (N/m^2^)	Muscle tone (Hz)	Stiffness (N/m^2^)
Left	3	12.48	179.06	12.82	184.17
	4	12.55	161.17	13.30	191.58
	3	14.01	213.89	14.05	216.33
	4	15.64	314.89	16.29	353.33
	3	17.12	389.94	16.63	387.58
Right	4	13.77	226.83	13.63	212.17
	3	15.49	302.50	15.95	321.42
	6	15.42	301.83	15.58	314.42
Bilateral	4	16.31	311.28	16.13	286.75
	3	17.96	406.72	18.84	430.92
	4	13.61	201.72	13.61	204.42
	4	20.67	497.50	21.19	515.58
	5	16.04	330.72	15.84	303.75
	3	19.51	438.17	18.66	424.75
	5	13.10	208.67	12.58	194.17

### Repeatability

The results of descriptive statistics indicated the mean differences in muscle tone and stiffness between the two scanning modes and between the two measurements were small. Tables 3 and 4 present the results of mean muscle tone and stiffness respectively. ICCs indicated excellent repeatability in triple scans and 5-scans mode for each lumbar level bilaterally. The confident interval of ICC were narrow in triple scans and 5-scans mode, indicating acceptable repeatability for both scanning modes to measure lumbar muscle tone and stiffness. SEM and SRD were smaller in triple scans than 5-scans mode for most levels when measuring muscle tone on the left. For muscle stiffness, the SEM and SRD were smaller in triple scans than 5-scans mode on the left side. SEM and SRD were larger in 5-scans mode than in triple scans mode on the right side. Tables 5 and 6 present the reliability indices of ICC, SEM and SRD for muscle tone and stiffness respectively.

**Table 3:** Mean measurements of muscle tone

Triple scans				5-scans	
Side	Levels	1st measurement (Hz)	2nd measurement (Hz)	1st measurement (Hz)	2nd measurement (Hz)
Left	L3	16.14 (2.38)	15.87 (2.24)	16.01 (2.28)	16.25 (2.28)
	L4	15.75 (2.58)	15.58 (2.50)	15.73 (2.56)	15.93 (2.69)
	L5	15.13 (2.80)	14.93 (2.58)	15.23 (2.85)	15.42 (3.12)
Right	L3	15.83 (2.25)	16.15 (2.41)	16.08 (2.12)	16.24 (2.28)
	L4	15.55 (2.45)	15.79 (2.47)	15.82 (2.42)	15.93 (2.69)
	L5	14.95 (2.70)	15.18 (2.81)	15.13 (2.72)	15.42 (3.12)

**Table 4:** Mean measurements of muscle stiffness

Triple scans				5-scans	
Side	Levels	1st measurement (N/m)	2nd measurement (N/m)	1st measurement (N/m)	2nd measurement (N/m)
Left	L3	328.06 (102.04)	317.53 (94.13)	325.07 (97.22)	328.47 (99.85)
	L4	304.13 (106.13)	299.67 (106.11)	305.53 (105.01)	315.07 (112.60)
	L5	272.47 (109.45)	267.60 (101.09)	278.13 (111.06)	281.40 (119.780
Right	L3	316.80 (101.42)	318.80 (99.03)	324.47 (97.50)	328.47 (99.85)
	L4	305.27 (104.61)	307.73 (106.24)	313.47 (106.79)	315.07 (112.59)
	L5	270.93(107.02)	273.33 (108.54)	277.33 (107.92)	281.40 (119.78)

**Table 5:** Repeatability indices for muscle tone

Side	Levels	ICC		95% CI		SEM (Hz)		SRD (Hz)	
		Triple	5-scans	Triple	5-scans	Triple	5-scans	Triple	5-scans
Left	L3	0.987	0.994	0.951–0.996	0.981–0.998	0.05	0.07	0.60	0.75
	L4	0.989	0.994	0.968–0.996	0.982–0.993	0.06	0.04	0.66	0.55
	L5	0.987	0.990	0.971–0.998	0.993–0.999	0.03	0.03	0.46	0.46
Right	L3	0.951	0.990	0.902–0.976	0.956–0.995	0.10	0.11	0.86	0.92
	L4	0.993	0.986	0.967–0.998	0.959–0.995	0.03	0.07	0.51	0.74
	L5	0.992	0.986	0.974–0.998	0.957–0.996	0.04	0.07	0.54	0.74

**Table 6:** Repeatability indices for muscle stiffness

Side	Levels	ICC		95% CI		SEM (N/m)		SRD (N/m)	
		Triple	5-scans	Triple	5-scans	Triple	5-scans	Triple	5-scans
Left	L3	0.987	0.994	0.962–0.995	0.960–0.990	1.70	1.16	3.61	2.98
	L4	0.993	0.998	0.979–0.998	0.993–0.999	1.82	0.97	3.73	2.74
	L5	0.983	0.996	0.963–0.996	0.987–0.999	2.39	0.91	4.29	2.65
Right	L3	0.994	0.988	0.960–0.998	0.964–0.996	2.27	2.42	4.18	4.31
	L4	0.990	0.987	0.971–0.997	0.962–0.996	2.15	2.91	4.06	4.73
	L5	0.995	0.991	0.986–0.998	0.974–0.997	1.08	2.05	2.88	3.97

Systematic bias was not observed in the Bland and Altman analysis of pooled muscle tone and stiffness on bilateral side when assessed by two scanning modes. The 95% limits of agreement for bilateral muscle tone and stiffness recorded by the two different scanning modes are presented in Table 7.

**Table 7:** Bland and Altman 95% limits of agreement for pooled muscle tone and stiffness

	Left						Right					
	Triple scans			5-scans			Triple scans			5-scans		
	Mean diff	Lower LOA	Upper LOA	Mean diff	Lower LOA	Upper LOA	Mean diff	Lower LOA	Upper LOA	Mean diff	Lower LOA	Upper LOA
Tone (Hz)	−0.90	−0.83	1.26	−0.80	−0.61	0.75	−1.60	−1.17	0.64	−2.00	−1.36	0.98
Stiffness (N/m)	−31.00	−32.72	45.97	−67.20	−42.77	59.52	−45.40	−41.55	36.98	−68.76	−48.50	42.05

### Correlation analysis

Spearman’s Rank correlation analysis indicated significant high correlation between muscle tone and disability level (*r* = 0.80, *p* < 0.05)., and between muscle stiffness and disability level (*r* = 0.81, *p* < 0.05). No significant correlation was observed between muscle tone and NPRS (*r* = − 0.24, *p* > 0.05) and between muscle stiffness and NPRS (*r* = − 0.21, *p* > 0.05).

## Discussions

This study aimed to explore if paraspinal muscle mechanical properties of tone and stiffness were repeatable parameters when measured in a clinical setting. The study also explored if different scanning modes had any impact on the reproducibility of the measures of muscle properties. The findings of the study indicated that the measures of muscle tone and stiffness were repeatable parameters when measured within a single session. No difference in reproducibility of the measures of mechanical muscle properties was observed between the two scanning mode with different number of indentations.

### Intraclass correlation coefficient

ICC is an indication of the agreement and consistency between measurements. The higher the agreement, the closer the ICC value to 1. The present study observed ICC values of over 0.9 between measurements and between different scanning modes. This indicated that muscle tone and stiffness were repeatable parameters and might be sufficiently reliable to be used as outcome measures. Eriksson et al., [39] assessed the repeatability of muscle stiffness of the lumbar spine muscles by palpation (feeling the resistance coming off the soft tissue by hand) and graded the stiffness with the category very low, low, middle high and very high. Results indicated lumbar muscles stiffness were repeatable (Kappa coefficient 0.82 *p* < 0.04) on a five categories scoring system. This finding is consistent with the findings of the present study that myotonometer reported acceptable repeatability when assessed by ICC. In addition, different number of indentations did not appear to affect the consistency between measurements. Thus, clinicians may be able to choose a lower number scanning mode for time-effectiveness purpose.

### SEM and SRD

Although the results of ICC analysis demonstrated acceptable reliability, however, ICC on its own was insufficient to assess repeatability as it only measures the magnitude of relative agreement and has the limitation of being influenced by the variance within the dataset [38]. Indices that are in absolute values are therefore essential to complement the result of ICC. SEM and SRD assess the degree variation between measurements for individuals [40]. These indices are in absolute values that enable comparisons between studies. SEM refers to the repeating error around the mean. A small SEM value suggests small amount of error spread around the mean. SRD is the smallest change that could be interpreted as real change. The indices of SEM and SRD for muscle tone and stiffness were similar between the two scanning modes, suggesting that the higher number of indentations were unlikely to improve the repeatability of muscle tone and stiffness in this population. The SEM and SRD observed in the present study were lower than the values reported for peripheral muscles in people with different pathological conditions or in healthy individuals. The SEM values reported in people with spinal cord injury were 0.98 Hz and 0.86 Hz for muscle tone, and 16.78 N/m and 20.36 N/m for right and left rectus femoris respectively [41]. When comparing with the repeatability of quadriceps muscle recorded in healthy individuals (SEM 0.7 Hz, SRD 1.9 Hz for muscle tone; SEM 10.7 N/m, SRD 29.7 N/m) [42], both SEM and SRD were lower than those published values, suggesting that lumbar muscle tone and stiffness are repeatable parameters. There is insufficient data to suggest if the observed repeatability may be appropriate to be used as outcome measure for intervention for this population. A recently published study indicated that spinal exercise may reduce muscle tone by approximately 1.5 Hz and 1.1 Hz, and reduce muscle stiffness by approximately 50 N/m and 30 N/m at L_3_ and L_5_ levels respectively [13]. The data from this study give some support that the SRD values observed in this study may be clinically acceptable.

### Bland & Altman analysis

Bland & Altman analysis is a statistical method to identify systematic bias. The 95% limits of agreement give an indication of whether the error range may be clinically accepted [40]. The Bland and Altman plots indicated the reproducibility of the measures of muscle tone and stiffness reduced when the measurements increased. Van Deun also reported consistent finding where reproducibility of the measures of muscle tone of bicep brachii were low when muscle tone value was high [6]. It was suggested that using a higher number of indentations or a second series to record muscle parameters may be improve reproducibility. The present study however did not observe any improvement in reproducibility and the same tendency was present in triple and 5-scans mode. The key difference between the present study and the study by Van Deun was that paratonia were confirmed by Paratonia Assessment Instrument. This study did not utilize other instrument to confirm mechanical properties of paraspinal muscles. Thus, no firm conclusion could be drawn to confirm if using higher number of indentations would improve reproducibility.

#### Mechanical properties of muscles and clinical measures

This study did not observe a significant association between intensity of pain and mechanical properties of muscles. Existing literature suggests that high muscle tone may contribute to chronic pain [10]. However, evidence that demonstrate the association of pain intensity with muscle mechanical properties of muscles in the LBP population was scarce. Contradictory evidence was also reported in a recent study that investigated muscle tone using EMG. The study conducted by Lothe et al., [43] reported no significant difference in muscle activity between people with low back pain when compared to pain free participants. Other study provided evidence to support that people with low back pain have different muscle activation pattern among the synergy muscles groups [44]. It is therefore unlikely that muscle properties might not be associated with pain level at rest but more related to pain elicited during movement. This is given some support by the association between mechanical properties of muscle and disability level observed in the present study. Study that assessed stiffness of multifidus using shearwave elastography reported significant difference between people with low back pain and asymptomatic individuals [45]. This finding reflect a deficit in activation of the multifidus and might subsequently contribute to functional disability. No firm conclusion could be drawn from the results of the present study due to the small sample size and the same pain range of the sample population.

### Limitations

This study did not control other factors that might influence muscle tone and stiffness, such as local subcutaneous soft tissue, ambient and body temperature. In addition, the exact state of the muscle during data collection session was not quantified by other mean. Therefore, it could not be ascertain that muscle structures were in a resting state. However, this should have minimal impact on the repeatability analysis since the analysis were based on within participant’s data rather than between participants. There was limited information regarding the recovery time for a muscle to return to its previous state. It was therefore unclear if the 15-min gap between each recording series was sufficient to enable muscle to relax and return to previous state. This may lead to underestimation of repeatability. The study did not conduct subgroup analysis based on pain location due to the uneven distribution and small sample size of the subgroups. Interferential statistics based on subgroups were therefore not meaningful. Further study with sufficiently large sample size to enable such subgroup analysis is recommended to further investigate the association between intensity of pain and mechanical properties of muscles. The lack of association was partially due to the study design that was set out to assess the relationship between pain intensity and muscle tone but the repeatability between measurements in different scanning mode by two assessors. The sample population reported to have pain score between 3 to 6. The small pain range is likely to result in low correlation coefficient from the statistically point of view as indicated in published literature [38]. This study had not recorded the history of clinical intervention that participants may have received. It was unclear if the 2 weeks wash out period was sufficient to wash out any potential effects on mechanical properties of muscles induced by interventions. However, this would have minimum effects on the results and conclusions of the present study since it primarily concerned the repeatability of parameters. This study did not specifically attempt to blind the assessor as it was unlikely that the results would be influenced by the assessors since they were only required to position the probe perpendicular to the skin surface of the test site) and had minimum involvement during the actual recording process. Myotonometer itself is not without limitation. It measures not only the mechanical properties of muscles but also the properties of other tissues, including skin elasticity and subcutaneous tissues. Despite the limitation of the technology, it should not impact the reliability analysis since the primary objective of the study was to compare between the two measurements. Further study to investigate the exact spinal tissue that is probed by the myotonometer is recommended to improve the clinical application of the device.

## Conclusions

This study found that muscle tone and stiffness were repeatable parameters when measured by myotonometer. The reproducibility of muscle tone and stiffness did not appear to differ between the two scanning modes with different number of indentations. Muscle tone and stiffness measured by myotonometer may therefore be reliable as outcome measures to assess intervention induced changes.

## Data Availability

The dataset supporting the conclusions of this article is available from the authors upon request.

## Abbreviations

BMI: Body mass index
EMG: Electromyography
Hz: Hertz
ICC: Intraclass correlation coefficient
LBP: Low back pain
LOA: Limits of agreement
N/m: Newton/meter
NPRS: Numerical Pain Rating Scale
ODI: Osewstry Disability Index
SEM: Standard error of measurement
SRD: Smallest real difference
